# 3-Chloro-*N*-(3-chloro­phen­yl)benzamide

**DOI:** 10.1107/S1600536808012099

**Published:** 2008-04-30

**Authors:** B. Thimme Gowda, Sabine Foro, B. P. Sowmya, Hartmut Fuess

**Affiliations:** a Department of Chemistry, Mangalore University, Mangalagangotri 574 199, Mangalore, India; b Institute of Materials Science, Darmstadt University of Technology, Petersenstrasse 23, D-64287 Darmstadt, Germany

## Abstract

In the crystal structure of the title compound, C_13_H_9_Cl_2_NO, the N—H and C=O bonds are *anti* to each other in the two independent mol­ecules. In one mol­ecule, the N—H bond is *syn* to the *meta*-chloro group of the attached ring; it is *anti* in the other mol­ecule. This relationship is also observed between the C=O bond and the *meta*-chloro substituent of its attached ring. The amide –NHCO– group makes dihedral angles of 31.5 (4) and 34.7 (3)° with the aniline rings; it makes dihedral angles of 37.4 (3) and 37.2 (3)° with the benzoyl rings. The two rings are nearly coplanar, with dihedral angles of 9.1 (2) and 7.3 (3)° in the two independent mol­ecules. Adjacent mol­ecules are linked into infinite chains through N—H⋯O hydrogen bonds.

## Related literature

For background literature, see: Gowda *et al.* (2003 ▶, 2007 ▶, 2008 ▶).
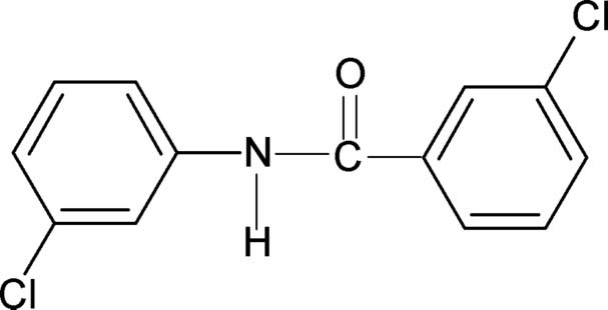


## Experimental

### 

#### Crystal data

C_13_H_9_Cl_2_NO
*M* *_r_* = 266.11
Monoclinic, 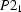

*a* = 8.577 (1) Å
*b* = 13.551 (1) Å
*c* = 10.357 (1) Å
β = 93.04 (1)°
*V* = 1202.1 (2) Å^3^
*Z* = 4
Cu *K*α radiation
μ = 4.70 mm^−1^
*T* = 296 (2) K
0.60 × 0.28 × 0.23 mm

#### Data collection

Enraf–Nonius CAD-4 diffractometer
Absorption correction: ψ scan (North *et al.*, 1968 ▶) *T*_min_ = 0.265, *T*_max_ = 0.341
3088 measured reflections
2249 independent reflections
2165 reflections with *I* > 2˘*I*)
*R*_int_ = 0.022
3 standard reflections frequency: 120 min intensity decay: none

#### Refinement

*R*[*F* ^2^ > 2σ(*F* ^2^)] = 0.046
*wR*(*F* ^2^) = 0.131
*S* = 1.10
2249 reflections
308 parameters
1 restraint
H-atom parameters constrained
Δρ_max_ = 0.37 e Å^−3^
Δρ_min_ = −0.49 e Å^−3^
Absolute structure: Flack (1983 ▶), no Friedel pairs
Flack parameter: 0.07 (2)

### 

Data collection: *CAD-4-PC* (Enraf–Nonius, 1996 ▶); cell refinement: *CAD-4-PC*; data reduction: *REDU4* (Stoe & Cie, 1987 ▶); program(s) used to solve structure: *SHELXS97* (Sheldrick, 2008 ▶); program(s) used to refine structure: *SHELXL97* (Sheldrick, 2008 ▶); molecular graphics: *PLATON* (Spek, 2003 ▶); software used to prepare material for publication: *SHELXL97*.

## Supplementary Material

Crystal structure: contains datablocks I, global. DOI: 10.1107/S1600536808012099/ng2449sup1.cif

Structure factors: contains datablocks I. DOI: 10.1107/S1600536808012099/ng2449Isup2.hkl

Additional supplementary materials:  crystallographic information; 3D view; checkCIF report

## Figures and Tables

**Table 1:** Hydrogen-bond geometry (Å, °)

*D*—H⋯*A*	*D*—H	H⋯*A*	*D*⋯*A*	*D*—H⋯*A*
N1—H1n⋯O2^i^	0.86	2.05	2.877 (4)	162
N2—H2n⋯O1	0.86	2.06	2.884 (5)	161

Symmetry code: (i) 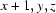.

**Fig. 1.**
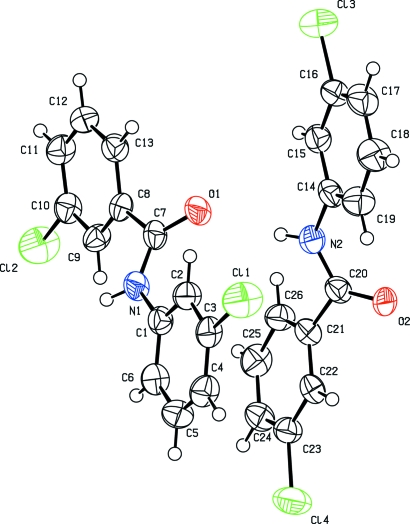
Molecular structure of the title compound, showing the atom labeling. Displacement ellipsoids are drawn at the 50% probability level.

**Fig. 2.**
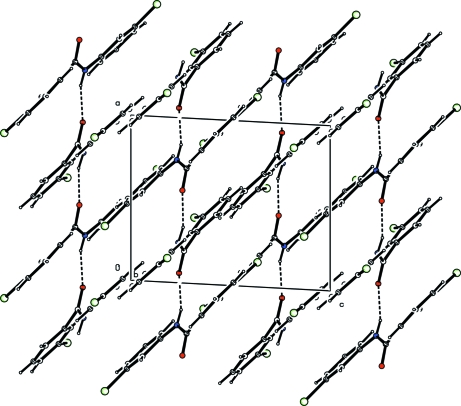
Molecular packing of the title compound with hydrogen bonding shown as dashed lines..

## supplementary crystallographic information

### Comment

As part of a study of the substituent effects on the structures of N-aromatic amides,in the present work, the structure of 3-Chloro-*N*-(3-chlorophenyl)- benzamide(N3CP3CBA) has been determined(Gowda *et al.*, 2003; 2007, 2008). In the structure of N3CP3CBA (Fig. 1),the conformations of the N—H and C=O bonds are *anti* to each other. The asymmetric unit of the structure contains two molecules. In one of the molecules, the conformation of the N—H bond is *syn* to the *meta*-chloro group in the aniline ring and *anti* to each other in the other molecule. Similar conformations were observed between the C=O bond and *meta*-chloro substituent in the benzoyl ring. This is in contrast to the single molecule observed in the asymmetric unit of 2-chloro-*N*-(2-Chlorophenyl)-benzamide (N2CP2CBA) and *syn* conformation of the N—H bond to the *ortho*-chloro substituent in the aniline ring and the C=O bond to the *ortho*-chloro substituent in the benzoyl ring (Gowda *et al.*, 2007). The bond parameters in N3CP3CBA are similar to those in *N*-(3-chlorophenyl)-benzamide, N2CP2CBA and other benzanilides (Gowda *et al.*, 2003; 2008). The amide group,-NHCO– makes the dihedral angles of 31.5 (4), 37.4 (3)° (molecule 1) and 34.7 (3), 37.2 (3)° (molecule 2) with the aniline and benzoyl rings, respectively, while those between the benzoyl and aniline rings are 9.1 (2)° and 7.3 (3)° in the molecules 1 and 2, respectively. The packing diagram of N3CP3CBA molecules showing the hydrogen bonds N1—H1N···O2 and N2—H2N···O1 (Table 1) involved in the formation of molecular chains is given in Fig. 2.

### Experimental

The title compound was prepared according to the literature method (Gowda *et al.*, 2003). The purity of the compound was checked by determining its melting point. It was characterized by recording its infrared and NMR spectra. Single crystals of the title compound were obtained from an ethanolic solution and used for X-ray diffraction studies at room temperature.

### Refinement

The H atoms were positioned with idealized geometry using a riding model with C—H = 0.93–0.96 Å, N—H = 0.86 Å. All H atoms were refined with isotropic displacement parameters (set to 1.2 times of the *U*_eq_ of the parent atom).

### Crystal data

**Table:**

C_13_H_9_Cl_2_NO	*F*_000_ = 544
*M**_r_* = 266.11	*D*_x_ = 1.470 Mg m^−^^3^
Monoclinic, *P*2_1_	Cu *K*α radiation λ = 1.54180 Å
Hall symbol: P 2yb	Cell parameters from 25 reflections
*a* = 8.577 (1) Å	θ = 6.5–27.0º
*b* = 13.551 (1) Å	µ = 4.70 mm^−^^1^
*c* = 10.357 (1) Å	*T* = 296 (2) K
β = 93.04 (1)º	Thick needle, colourless
*V* = 1202.1 (2) Å^3^	0.60 × 0.28 × 0.23 mm
*Z* = 4	

### Data collection

**Table:**

Enraf–Nonius CAD-4 diffractometer	*R*_int_ = 0.022
Radiation source: medium-focus sealed tube	θ_max_ = 67.0º
Monochromator: graphite	θ_min_ = 4.3º
*T* = 296(2) K	*h* = −10→3
ω–2θ scans	*k* = −16→0
Absorption correction: ψ scan(North *et al.*, 1968)	*l* = −12→12
*T*_min_ = 0.265, *T*_max_ = 0.341	3 standard reflections
3088 measured reflections	every 120 min
2249 independent reflections	intensity decay: none
2165 reflections with *I* > 2˘*I*)	

### Refinement

**Table:**

Refinement on *F*^2^	Hydrogen site location: inferred from neighbouring sites
Least-squares matrix: full	H-atom parameters constrained
*R*[*F*^2^ > 2σ(*F*^2^)] = 0.046	*w* = 1/[σ^2^(*F*_o_^2^) + (0.0835*P*)^2^ + 0.4755*P*] where *P* = (*F*_o_^2^ + 2*F*_c_^2^)/3
*wR*(*F*^2^) = 0.131	(Δ/σ)_max_ = 0.010
*S* = 1.10	Δρ_max_ = 0.37 e Å^−^^3^
2249 reflections	Δρ_min_ = −0.49 e Å^−^^3^
308 parameters	Extinction correction: SHELXL97 (Sheldrick, 2008), Fc^*^=kFc[1+0.001xFc^2^λ^3^/sin(2θ)]^-1/4^
1 restraint	Extinction coefficient: 0.0062 (9)
Primary atom site location: structure-invariant direct methods	Absolute structure: No Flack (1983), no Friedel pairs
Secondary atom site location: difference Fourier map	Flack parameter: 0.07 (2)

### Special details

**Table:**

Geometry. All e.s.d.'s (except the e.s.d. in the dihedral angle between two l.s. planes) are estimated using the full covariance matrix. The cell e.s.d.'s are taken into account individually in the estimation of e.s.d.'s in distances, angles and torsion angles; correlations between e.s.d.'s in cell parameters are only used when they are defined by crystal symmetry. An approximate (isotropic) treatment of cell e.s.d.'s is used for estimating e.s.d.'s involving l.s. planes.
Refinement. Refinement of *F*^2^ against ALL reflections. The weighted *R*-factor *wR* and goodness of fit *S* are based on *F*^2^, conventional *R*-factors *R* are based on *F*, with *F* set to zero for negative *F*^2^. The threshold expression of *F*^2^ > σ(*F*^2^) is used only for calculating *R*-factors(gt) *etc*. and is not relevant to the choice of reflections for refinement. *R*-factors based on *F*^2^ are statistically about twice as large as those based on *F*, and *R*- factors based on ALL data will be even larger.

### Fractional atomic coordinates and isotropic or equivalent isotropic displacement parameters (Å^2^)

**Table:**

	*x*	*y*	*z*	*U*_iso_*/*U*_eq_	
Cl1	0.32030 (17)	−0.00289 (12)	−0.13075 (14)	0.0740 (5)	
Cl2	1.14912 (18)	0.14458 (13)	0.63527 (15)	0.0799 (5)	
O1	0.5608 (3)	0.1667 (2)	0.2587 (3)	0.0503 (8)	
N1	0.7425 (4)	0.0481 (3)	0.2308 (4)	0.0460 (8)	
H1N	0.8369	0.0308	0.2520	0.055*	
C1	0.6552 (5)	−0.0173 (3)	0.1482 (4)	0.0431 (10)	
C2	0.5418 (5)	0.0182 (4)	0.0581 (4)	0.0456 (10)	
H2	0.5206	0.0853	0.0511	0.055*	
C3	0.4622 (5)	−0.0494 (4)	−0.0200 (4)	0.0445 (10)	
C4	0.4926 (6)	−0.1489 (4)	−0.0152 (5)	0.0515 (11)	
H4	0.4385	−0.1926	−0.0703	0.062*	
C5	0.6063 (6)	−0.1817 (4)	0.0742 (4)	0.0535 (11)	
H5	0.6279	−0.2489	0.0807	0.064*	
C6	0.6879 (5)	−0.1163 (4)	0.1537 (4)	0.0488 (10)	
H6	0.7660	−0.1394	0.2117	0.059*	
C7	0.6948 (4)	0.1331 (3)	0.2792 (4)	0.0422 (9)	
C8	0.8119 (4)	0.1892 (4)	0.3622 (4)	0.0393 (9)	
C9	0.9189 (5)	0.1425 (4)	0.4483 (4)	0.0441 (9)	
H9	0.9228	0.0741	0.4541	0.053*	
C10	1.0191 (5)	0.2006 (4)	0.5251 (4)	0.0459 (10)	
C11	1.0193 (6)	0.3013 (4)	0.5145 (5)	0.0485 (10)	
H11	1.0898	0.3389	0.5649	0.058*	
C12	0.9142 (5)	0.3464 (4)	0.4286 (5)	0.0496 (11)	
H12	0.9139	0.4148	0.4208	0.060*	
C13	0.8099 (5)	0.2910 (4)	0.3546 (4)	0.0456 (10)	
H13	0.7371	0.3222	0.2987	0.055*	
Cl3	0.1457 (2)	0.46405 (11)	0.18206 (16)	0.0753 (4)	
Cl4	0.39972 (18)	−0.34554 (10)	0.31885 (16)	0.0731 (4)	
O2	0.0590 (3)	−0.0229 (2)	0.2416 (3)	0.0517 (8)	
N2	0.2432 (4)	0.0962 (3)	0.2377 (4)	0.0485 (9)	
H2N	0.3378	0.1108	0.2621	0.058*	
C14	0.1548 (5)	0.1709 (3)	0.1702 (4)	0.0428 (10)	
C15	0.1861 (5)	0.2671 (4)	0.2042 (4)	0.0465 (10)	
H15	0.2607	0.2819	0.2697	0.056*	
C16	0.1049 (6)	0.3412 (4)	0.1395 (5)	0.0502 (11)	
C17	−0.0064 (6)	0.3217 (4)	0.0413 (5)	0.0576 (12)	
H17	−0.0604	0.3727	−0.0011	0.069*	
C18	−0.0353 (6)	0.2251 (4)	0.0079 (5)	0.0604 (13)	
H18	−0.1089	0.2106	−0.0585	0.072*	
C19	0.0435 (6)	0.1493 (4)	0.0719 (4)	0.0532 (10)	
H19	0.0222	0.0841	0.0493	0.064*	
C20	0.1947 (5)	0.0069 (3)	0.2667 (4)	0.0433 (9)	
C21	0.3143 (5)	−0.0594 (3)	0.3333 (4)	0.0425 (9)	
C22	0.3057 (5)	−0.1578 (4)	0.3017 (4)	0.0425 (10)	
H22	0.2313	−0.1802	0.2400	0.051*	
C23	0.4090 (5)	−0.2227 (3)	0.3627 (4)	0.0464 (10)	
C24	0.5166 (5)	−0.1926 (4)	0.4561 (5)	0.0518 (11)	
H24	0.5846	−0.2378	0.4966	0.062*	
C25	0.5229 (6)	−0.0953 (4)	0.4891 (5)	0.0560 (12)	
H25	0.5951	−0.0742	0.5535	0.067*	
C26	0.4227 (5)	−0.0268 (4)	0.4277 (4)	0.0477 (10)	
H26	0.4286	0.0397	0.4498	0.057*	

### Atomic displacement parameters (Å^2^)

**Table:**

	*U*^11^	*U*^22^	*U*^33^	*U*^12^	*U*^13^	*U*^23^
Cl1	0.0702 (8)	0.0747 (9)	0.0728 (8)	−0.0055 (7)	−0.0370 (6)	0.0012 (7)
Cl2	0.0704 (8)	0.0792 (10)	0.0852 (10)	−0.0037 (8)	−0.0431 (7)	0.0120 (8)
O1	0.0270 (13)	0.052 (2)	0.0705 (19)	0.0027 (12)	−0.0092 (12)	−0.0101 (15)
N1	0.0294 (16)	0.051 (2)	0.056 (2)	0.0007 (15)	−0.0091 (14)	−0.0052 (18)
C1	0.0343 (19)	0.048 (3)	0.046 (2)	−0.0029 (18)	0.0001 (16)	−0.0051 (19)
C2	0.042 (2)	0.045 (2)	0.049 (2)	−0.0022 (18)	−0.0042 (17)	−0.0010 (19)
C3	0.034 (2)	0.056 (3)	0.042 (2)	−0.0049 (19)	−0.0059 (17)	−0.004 (2)
C4	0.047 (2)	0.059 (3)	0.048 (2)	−0.011 (2)	−0.0056 (19)	−0.009 (2)
C5	0.060 (3)	0.046 (3)	0.055 (3)	0.003 (2)	0.003 (2)	−0.006 (2)
C6	0.042 (2)	0.061 (3)	0.042 (2)	0.000 (2)	−0.0042 (17)	−0.001 (2)
C7	0.0293 (18)	0.050 (3)	0.047 (2)	−0.0013 (18)	−0.0030 (15)	0.0009 (19)
C8	0.0307 (18)	0.048 (2)	0.0391 (19)	−0.0065 (17)	−0.0021 (15)	−0.0006 (17)
C9	0.041 (2)	0.042 (2)	0.048 (2)	−0.0057 (19)	−0.0043 (17)	0.0012 (19)
C10	0.037 (2)	0.057 (3)	0.043 (2)	0.0004 (19)	−0.0072 (16)	0.002 (2)
C11	0.047 (2)	0.052 (3)	0.047 (2)	−0.010 (2)	−0.0028 (18)	−0.011 (2)
C12	0.049 (3)	0.041 (2)	0.058 (3)	−0.0035 (19)	0.003 (2)	−0.006 (2)
C13	0.039 (2)	0.049 (3)	0.049 (2)	0.0023 (18)	0.0013 (17)	0.002 (2)
Cl3	0.0863 (10)	0.0465 (7)	0.0935 (10)	0.0010 (7)	0.0074 (8)	0.0058 (7)
Cl4	0.0804 (8)	0.0452 (7)	0.0932 (10)	0.0119 (6)	0.0009 (7)	−0.0068 (7)
O2	0.0252 (13)	0.0485 (18)	0.080 (2)	−0.0024 (13)	−0.0067 (12)	0.0044 (16)
N2	0.0358 (19)	0.047 (2)	0.062 (2)	0.0023 (16)	−0.0041 (16)	0.0028 (19)
C14	0.0340 (19)	0.049 (3)	0.046 (2)	0.0101 (17)	0.0044 (16)	0.0074 (19)
C15	0.040 (2)	0.049 (3)	0.051 (2)	0.0023 (19)	0.0038 (18)	0.0041 (19)
C16	0.049 (2)	0.044 (3)	0.059 (3)	0.0066 (19)	0.009 (2)	0.005 (2)
C17	0.051 (3)	0.062 (3)	0.059 (3)	0.015 (2)	−0.004 (2)	0.015 (2)
C18	0.053 (3)	0.066 (3)	0.060 (3)	0.007 (2)	−0.015 (2)	0.008 (3)
C19	0.060 (3)	0.049 (2)	0.049 (2)	0.004 (2)	−0.0058 (19)	0.002 (2)
C20	0.038 (2)	0.044 (2)	0.048 (2)	0.0064 (18)	−0.0026 (16)	−0.0033 (19)
C21	0.039 (2)	0.045 (2)	0.043 (2)	0.0084 (18)	0.0011 (16)	0.0008 (18)
C22	0.0335 (19)	0.052 (3)	0.041 (2)	0.0070 (18)	−0.0013 (16)	−0.0019 (18)
C23	0.046 (2)	0.042 (2)	0.051 (2)	0.0043 (19)	0.0058 (18)	0.0039 (19)
C24	0.044 (2)	0.051 (3)	0.060 (3)	0.012 (2)	−0.005 (2)	0.012 (2)
C25	0.047 (3)	0.066 (3)	0.054 (2)	−0.004 (2)	−0.013 (2)	0.004 (2)
C26	0.050 (2)	0.040 (2)	0.052 (2)	0.002 (2)	−0.0029 (18)	0.000 (2)

### Geometric parameters (Å, °)

**Table:**

Cl1—C3	1.745 (4)	Cl3—C16	1.753 (5)
Cl2—C10	1.729 (4)	Cl4—C23	1.726 (5)
O1—C7	1.244 (5)	O2—C20	1.246 (5)
N1—C7	1.330 (6)	N2—C20	1.319 (6)
N1—C1	1.418 (5)	N2—C14	1.425 (5)
N1—H1N	0.8600	N2—H2N	0.8600
C1—C6	1.370 (7)	C14—C15	1.373 (7)
C1—C2	1.397 (6)	C14—C19	1.390 (6)
C2—C3	1.379 (6)	C15—C16	1.376 (7)
C2—H2	0.9300	C15—H15	0.9300
C3—C4	1.373 (7)	C16—C17	1.383 (7)
C4—C5	1.383 (7)	C17—C18	1.373 (8)
C4—H4	0.9300	C17—H17	0.9300
C5—C6	1.376 (7)	C18—C19	1.380 (7)
C5—H5	0.9300	C18—H18	0.9300
C6—H6	0.9300	C19—H19	0.9300
C7—C8	1.493 (6)	C20—C21	1.503 (6)
C8—C13	1.382 (6)	C21—C22	1.374 (6)
C8—C9	1.397 (6)	C21—C26	1.386 (6)
C9—C10	1.385 (6)	C22—C23	1.378 (6)
C9—H9	0.9300	C22—H22	0.9300
C10—C11	1.369 (7)	C23—C24	1.363 (7)
C11—C12	1.375 (7)	C24—C25	1.364 (8)
C11—H11	0.9300	C24—H24	0.9300
C12—C13	1.372 (6)	C25—C26	1.395 (7)
C12—H12	0.9300	C25—H25	0.9300
C13—H13	0.9300	C26—H26	0.9300
			
C7—N1—C1	127.3 (4)	C20—N2—C14	126.6 (4)
C7—N1—H1N	116.3	C20—N2—H2N	116.7
C1—N1—H1N	116.3	C14—N2—H2N	116.7
C6—C1—C2	119.9 (4)	C15—C14—C19	120.3 (4)
C6—C1—N1	119.1 (4)	C15—C14—N2	117.2 (4)
C2—C1—N1	121.0 (4)	C19—C14—N2	122.5 (4)
C3—C2—C1	117.9 (4)	C14—C15—C16	118.8 (4)
C3—C2—H2	121.0	C14—C15—H15	120.6
C1—C2—H2	121.0	C16—C15—H15	120.6
C4—C3—C2	122.9 (5)	C15—C16—C17	122.0 (5)
C4—C3—Cl1	120.2 (4)	C15—C16—Cl3	118.8 (4)
C2—C3—Cl1	116.9 (4)	C17—C16—Cl3	119.2 (4)
C3—C4—C5	117.8 (4)	C18—C17—C16	118.4 (5)
C3—C4—H4	121.1	C18—C17—H17	120.8
C5—C4—H4	121.1	C16—C17—H17	120.8
C6—C5—C4	120.7 (5)	C17—C18—C19	120.8 (5)
C6—C5—H5	119.6	C17—C18—H18	119.6
C4—C5—H5	119.6	C19—C18—H18	119.6
C1—C6—C5	120.6 (4)	C18—C19—C14	119.7 (5)
C1—C6—H6	119.7	C18—C19—H19	120.2
C5—C6—H6	119.7	C14—C19—H19	120.2
O1—C7—N1	123.5 (4)	O2—C20—N2	123.5 (4)
O1—C7—C8	120.0 (4)	O2—C20—C21	120.6 (4)
N1—C7—C8	116.5 (4)	N2—C20—C21	115.9 (4)
C13—C8—C9	119.6 (4)	C22—C21—C26	120.2 (4)
C13—C8—C7	118.0 (4)	C22—C21—C20	116.3 (4)
C9—C8—C7	122.4 (4)	C26—C21—C20	123.3 (4)
C10—C9—C8	118.4 (4)	C21—C22—C23	119.0 (4)
C10—C9—H9	120.8	C21—C22—H22	120.5
C8—C9—H9	120.8	C23—C22—H22	120.5
C11—C10—C9	121.6 (4)	C24—C23—C22	121.9 (5)
C11—C10—Cl2	119.2 (4)	C24—C23—Cl4	119.7 (4)
C9—C10—Cl2	119.2 (4)	C22—C23—Cl4	118.4 (4)
C10—C11—C12	119.4 (5)	C25—C24—C23	118.9 (4)
C10—C11—H11	120.3	C25—C24—H24	120.5
C12—C11—H11	120.3	C23—C24—H24	120.5
C13—C12—C11	120.3 (5)	C24—C25—C26	120.9 (5)
C13—C12—H12	119.9	C24—C25—H25	119.5
C11—C12—H12	119.9	C26—C25—H25	119.5
C12—C13—C8	120.6 (4)	C21—C26—C25	118.9 (5)
C12—C13—H13	119.7	C21—C26—H26	120.5
C8—C13—H13	119.7	C25—C26—H26	120.5
			
C7—N1—C1—C6	−150.4 (5)	C20—N2—C14—C15	147.2 (5)
C7—N1—C1—C2	32.5 (7)	C20—N2—C14—C19	−34.2 (7)
C6—C1—C2—C3	2.2 (6)	C19—C14—C15—C16	0.3 (6)
N1—C1—C2—C3	179.4 (4)	N2—C14—C15—C16	178.9 (4)
C1—C2—C3—C4	−1.8 (7)	C14—C15—C16—C17	−0.3 (7)
C1—C2—C3—Cl1	179.6 (3)	C14—C15—C16—Cl3	−179.6 (3)
C2—C3—C4—C5	1.3 (7)	C15—C16—C17—C18	−0.3 (8)
Cl1—C3—C4—C5	179.9 (4)	Cl3—C16—C17—C18	179.1 (4)
C3—C4—C5—C6	−1.2 (7)	C16—C17—C18—C19	0.8 (8)
C2—C1—C6—C5	−2.3 (7)	C17—C18—C19—C14	−0.8 (8)
N1—C1—C6—C5	−179.5 (4)	C15—C14—C19—C18	0.2 (7)
C4—C5—C6—C1	1.7 (7)	N2—C14—C19—C18	−178.3 (4)
C1—N1—C7—O1	1.2 (7)	C14—N2—C20—O2	−2.7 (7)
C1—N1—C7—C8	−178.8 (4)	C14—N2—C20—C21	177.2 (4)
O1—C7—C8—C13	−36.2 (6)	O2—C20—C21—C22	35.5 (6)
N1—C7—C8—C13	143.8 (4)	N2—C20—C21—C22	−144.4 (4)
O1—C7—C8—C9	142.1 (4)	O2—C20—C21—C26	−140.5 (4)
N1—C7—C8—C9	−37.9 (6)	N2—C20—C21—C26	39.5 (6)
C13—C8—C9—C10	0.8 (6)	C26—C21—C22—C23	−1.7 (6)
C7—C8—C9—C10	−177.5 (4)	C20—C21—C22—C23	−177.9 (4)
C8—C9—C10—C11	−2.6 (7)	C21—C22—C23—C24	1.9 (7)
C8—C9—C10—Cl2	178.4 (3)	C21—C22—C23—Cl4	−178.4 (3)
C9—C10—C11—C12	2.1 (8)	C22—C23—C24—C25	−0.6 (7)
Cl2—C10—C11—C12	−178.8 (4)	Cl4—C23—C24—C25	179.7 (4)
C10—C11—C12—C13	0.2 (7)	C23—C24—C25—C26	−0.8 (8)
C11—C12—C13—C8	−2.0 (7)	C22—C21—C26—C25	0.3 (6)
C9—C8—C13—C12	1.5 (7)	C20—C21—C26—C25	176.3 (4)
C7—C8—C13—C12	179.8 (4)	C24—C25—C26—C21	0.9 (7)

### Hydrogen-bond geometry (Å, °)

**Table:**

*D*—H···*A*	*D*—H	H···*A*	*D*···*A*	*D*—H···*A*
N1—H1n···O2^i^	0.86	2.05	2.877 (4)	161.7
N2—H2n···O1	0.86	2.06	2.884 (5)	160.5

Symmetry codes: (i) *x*+1, *y*, *z*.

## References

[bb1] Enraf–Nonius (1996). *CAD-4-PC* Enraf–Nonius, Delft, The Netherlands.

[bb2] Flack, H. D. (1983). *Acta Cryst.* A**39**, 876–881.

[bb3] Gowda, B. T., Foro, S., Sowmya, B. P. & Fuess, H. (2007). *Acta Cryst.* E**63**, o3789.

[bb4] Gowda, B. T., Jyothi, K., Paulus, H. & Fuess, H. (2003). *Z. Naturforsch. Teil A*, **58**, 225–230.

[bb5] Gowda, B. T., Tokarčík, M., Kožíšek, J., Sowmya, B. P. & Fuess, H. (2008). *Acta Cryst.* E**64**, o462. 10.1107/S1600536808001311 PMC2960316 21201488

[bb6] North, A. C. T., Phillips, D. C. & Mathews, F. S. (1968). *Acta Cryst.* A**24**, 351–359.

[bb7] Sheldrick, G. M. (2008). *Acta Cryst.* A**64**, 112–122. 10.1107/S0108767307043930 18156677

[bb8] Spek, A. L. (2003). *J. Appl. Cryst.* **36**, 7–13.

[bb9] Stoe & Cie (1987). *REDU4* Stoe & Cie GmbH, Darmstadt, Germany.

